# Sensor Technologies for Non-Invasive Blood Glucose Monitoring

**DOI:** 10.3390/s25123591

**Published:** 2025-06-07

**Authors:** Jiale Shi, Raúl Fernández-García, Ignacio Gil

**Affiliations:** Department of Electronic Engineering, Universitat Politecnica de Catalunya, 08222 Barcelona, Spain; raul.fernandez-garcia@upc.edu (R.F.-G.); ignasi.gil@upc.edu (I.G.)

**Keywords:** diabetes, blood glucose level, sensor technologies, non-invasive, antenna-sensor, rigid, flexible, phantom, textile, SAR

## Abstract

Diabetes poses a significant global health challenge, underscoring the urgent need for accurate and continuous glucose monitoring technologies. This review provides a comprehensive analysis of both invasive and non-invasive sensor technologies, with a particular focus on antenna-sensors and their working principle. Key aspects, including the selection of substrates and conductive materials, fabrication techniques, and recent advancements in rigid and flexible antenna-sensor designs, are critically evaluated. Notably, textile antenna-sensors are gaining increasing attention due to their potential for seamless integration into daily clothing. Furthermore, the influence of the human body on antenna-sensor performance is examined, emphasizing the importance of human phantom simulation and fabrication for precise modeling and validation. Finally, this review highlights the current technical challenges in the development of flexible antenna-sensors and discusses their transformative potential in enabling next-generation, non-invasive, and patient-centric glucose monitoring solutions.

## 1. Introduction

The human body is characterized by many important physiological parameters, such as blood pressure, respiratory rate, and heart rate, which together reflect the health condition. Among these, the blood glucose level is a fundamental physiological parameter, indispensable for sustaining human health. As the primary energy source, glucose facilitates cellular metabolism and plays an essential role in regulating the function of vital organs [1]. Disruptions in glucose homeostasis can lead to serious complications, most notably diabetes.

Diabetes is characterized by chronic hyperglycemia due to impaired insulin secretion or insulin resistance [2,3]. It encompasses several major types—including type 1, type 2, and gestational diabetes, as well as rarer forms such as monogenic and secondary diabetes [4,5,6,7,8,9,10,11,12,13]. As the most prevalent endocrine disease, diabetes has become an increasing health burden worldwide, affecting individuals, families, and entire nations. According to the Diabetes Atlas published by the International Diabetes Federation in 2021 [14], the global prevalence of diabetes among adults aged 20 to 79 is currently 10.5%. Projections indicate that by 2045, this figure is expected to rise to 783 million people, indicating a very serious growth trend for the disease worldwide. Table 1 presents the estimated number for different regions.

Effective diabetes management encompasses several components, including patient education, dietary control [15], regular physical activity [16], and the monitoring of blood glucose levels. Among these, blood glucose monitoring is pivotal in optimizing treatment plans and preventing long-term complications. According to the established diagnostic criteria [17], a plasma glucose level of 200 mg/dL or higher during a 2 h oral glucose tolerance test indicates diabetes. Hence, reliable and accurate blood glucose monitoring technologies are essential to support effective disease management.

This review methodology was based on a thorough search of conference proceedings and full-text articles from a wide array of sources and databases, including Science Direct, MDPI, Springer, and IEEE Xplore. In Section 2, our literature search strategy was designed to encompass a wide array of sensor technologies, incorporating both invasive and non-invasive methodologies. Given the inherent diversity of these technologies, it was impractical to employ a uniform set of search terms across all inquiries. Instead, we adopted a targeted approach, selecting specific keywords that aligned with the underlying principles and operational mechanisms of each technology. For instance, terms such as “optical sensor,” “bioimpedance,” “reverse iontophoresis,” and “electromagnetic sensing” were utilized to reflect the unique characteristics of the respective methods. This customized search strategy facilitated a thorough and systematic collection of pertinent studies, ensuring comprehensive coverage across various technological domains. For Section 3, the keywords employed in each database were as follows: (rigid OR flexible OR textile) AND (blood glucose) AND (antenna OR electromagnetic OR microwave) AND (sensor). The preliminary search yielded 204 results. After screening and analyzing these findings to remove any duplicates, only 75 studies were deemed relevant. Among these, 12% were feasibility studies that explored the use of antenna-sensors for non-invasive glucose monitoring, 61.7% concentrated on rigid antenna-sensors for glucose measurement, and 21.3% examined flexible antenna-sensors for the same purpose.

The paper is organized as follows: Section 2 provides an in-depth examination of both invasive and non-invasive sensor technologies for blood glucose monitoring, as documented in the existing research. It evaluates their underlying principles, strengths, and drawbacks. Section 3 delves into the fundamental workings of antenna-sensors designed for blood glucose detection. It covers the substrate and conductive materials typically used for rigid and flexible antenna-sensors, along with the fabrication techniques and recent advancements. This section also highlights how the human body influences antenna-sensor performance and underscores the importance of simulating and creating human phantoms for testing. Finally, Section 4 consolidates the main insights, identifies the critical obstacles in developing flexible antenna-sensors, and suggests potential avenues for future research.

## 2. Common Sensor Technologies for Blood Glucose Monitoring

This section presents a review of the widely used invasive and non-invasive sensor technologies for blood glucose monitoring.

### 2.1. Invasive Sensors

Currently, the primary methods for monitoring blood glucose levels in medical settings and daily life are based on two invasive sensor technologies. The first is the Self-Monitoring Blood Glucose (SMBG) system, which assesses glucose levels in capillary blood. The second is the Continuous Glucose Monitoring (CGM) system, intended to measure glucose levels in interstitial fluid. These methods continue to be the favored solutions for effectively managing glucose levels.

#### 2.1.1. Self-Monitoring Blood Glucose

The SMBG system is a commonly used method for measuring blood glucose levels. It involves analyzing a small blood sample obtained from a finger prick. When the blood is applied to the reagent strip, glucose in the sample reacts with enzyme-based sensors, such as glucose oxidase, embedded in the strip. This reaction triggers a chemical process that generates an electrical signal. The SMBG system then interprets this signal to deliver an accurate glucose level reading.

SMBG systems are highly valued for their simplicity and reliability in daily glucose management. However, they are limited to providing single-point measurements, which means that they cannot capture the full spectrum of glucose fluctuations that occur throughout the day. Moreover, the necessity of frequent finger pricking can lead to discomfort for patients, potentially reducing patient compliance with regular monitoring routines [18].

A standout example of SMBG technology is Abbott’s FreeStyle Precision Neo glucose meter. This compact device quickly analyzes a tiny blood sample taken from the fingertip, providing precise readings in mere seconds. Beyond its core function, it boasts sophisticated features such as data storage and trend analysis, empowering users to monitor their glucose levels over time [19].

#### 2.1.2. Continuous Glucose Monitoring

CGM systems offer an innovative approach to monitoring blood glucose levels, utilizing a subcutaneous sensor that provides real-time data. These devices collect information at regular intervals, offering a comprehensive view of glucose trends throughout the day. This capability allows users to detect fluctuations and receive immediate alerts about potential hyperglycemia or hypoglycemia. CGM is especially advantageous for individuals experiencing frequent glucose level changes, as it provides a more continuous and detailed perspective compared to traditional methods of single-point sampling. However, the extensive adoption of CGM systems is frequently constrained by their high cost, and their accuracy may be affected during rapid glucose fluctuations, which could limit their effectiveness in certain scenarios [20].

A prominent example of CGM technology is the Dexcom G6 system. This advanced device features a subcutaneous sensor that can operate continuously for up to 10 days after implantation. It wirelessly transmits real-time glucose data to smart devices, enabling users to monitor their glucose levels conveniently [21]. Despite its advantages, the cost and accessibility of CGM systems present barriers for some patients, underscoring the need for further innovation and affordability in this field.

Although invasive sensor techniques, including SMBG and CGM, remain the clinical gold standard for glucose monitoring, they are not without significant limitations. One major issue is the long-term pain and discomfort associated with frequent skin punctures or the prolonged use of subcutaneous sensors. Another critical concern is the potential risk of transmitting blood-borne diseases, particularly in settings where blood glucose monitoring devices are shared among multiple patients. Improper sterilization or the reuse of lancets, needles, or other components can increase the likelihood of cross-contamination, posing a serious health risk. This issue is especially pertinent in clinical environments or regions with limited access to disposable medical supplies [22].

As a result, the advancement of non-invasive sensor technologies capable of reliably measuring blood glucose levels without causing physical harm has become a crucial focus in healthcare innovation. These advancements would significantly enhance patient comfort and reduce the drawbacks and risks associated with traditional invasive procedures. In recent decades, extensive research has been directed toward exploring various non-invasive methods for monitoring blood glucose.

### 2.2. Non-Invasive Sensors

Non-invasive blood glucose monitoring technology can be broadly categorized into two types: optical sensor technology and non-optical sensor technology. The former monitors blood glucose levels by utilizing the propagation characteristics of light, whereas the latter employs alternative physical or chemical principles for monitoring.

#### 2.2.1. Optical Sensor Technologies

Light is composed of photons that convey energy through wave-like motion. In the realm of sensing, light serves as a highly adaptable medium for transmitting information. By examining its various attributes—such as intensity, phase, frequency, polarization, and scattering properties—scientists can deduce the characteristics of a material indirectly. This concept underpins optical sensor technologies, which utilize the interplay between light and matter to gather critical insights without the need for direct contact or intrusive methods.

Optical sensor technologies can be categorized into various techniques based on their wavelength and interaction methods. These include near-infrared and mid-infrared spectroscopy, Fluorescence Spectroscopy, Raman Spectroscopy, and Optical Coherence Tomography, among others.

##### Near-Infrared/Mid-Infrared Spectroscopy

The wavelength range of near-infrared (NIR) spectroscopy spans from 780 to 2500 nm, making it a versatile tool for analyzing biological tissues. When infrared light passes through the skin, it interacts with molecules such as glucose and water, producing a unique spectral signature. However, one of the key challenges in NIR-based glucose monitoring is that glucose exhibits relatively weak absorption within the near-infrared range. Its absorption coefficient is significantly lower compared to other tissue components. As a result, the glucose signal is often obscured by background interference, necessitating the use of advanced noise reduction and signal processing algorithms to extract accurate glucose measurements [23].

For instance, a study by Li [24] presented an advanced nonlinear modeling algorithm. This method combines the elimination of minimum uninformative variables with kernel partial least squares, effectively reducing high-frequency noise and alleviating matrix background interference. In the simulated physiological solution sample experiment, the technology’s prediction root mean square error was 9.4 mg/dL. However, it does not consistently perform well with the complex spectral data produced by the human body, indicating a need for further improvement. Overall, this algorithm is anticipated to enhance the prediction accuracy of NIR spectroscopy in measuring blood glucose levels.

Mid-infrared (MIR) spectroscopy, which spans wavelengths from 2500 to 10,000 nm, holds significant promise for glucose detection due to its strong absorption affinity for glucose molecules. Unlike other methods, MIR spectroscopy is less prone to interference from surrounding tissue components, allowing for clearer and more precise spectral data related to glucose [25].

Yoshioka [26] made a pioneering contribution by developing an advanced attenuated total reflection absorption spectroscopy system based on MIR technology. He then conducted measurements at the lip surface, achieving a high correlation (R^2^ = 0.93) between the spectral features and capillary blood glucose concentrations. These results confirm the feasibility of MIR-based sensing in thin-skin regions. A major challenge of MIR is its limited penetration depth; under skin conditions, MIR light can only penetrate a few microns into the tissue [27]. This limitation complicates the efforts to measure the glucose levels in deeper tissues or through thicker skin.

Overall, while both NIR and MIR spectroscopy hold promise for non-invasive glucose monitoring, their practical applicability differs significantly. NIR offers greater tissue penetration but suffers from weak glucose-specific signals and high susceptibility to background interference. In contrast, MIR provides clearer spectral features, yet is constrained by the shallow penetration and greater hardware complexity. These complementary strengths and limitations suggest that future advancements may benefit from hybrid sensing strategies.

##### Fluorescence Spectroscopy

Fluorescence spectroscopy (FS) is a cutting-edge analytical method that relies on the fluorescence phenomenon. It involves illuminating biological tissues with ultraviolet light at precise frequencies and intensities. When the tissues are excited, specific molecules emit distinct fluorescence, which is closely related to their chemical composition and concentration. Since the fluorescence signature is unique to each fluorescent substance, FS becomes a valuable tool for gaining insights into blood glucose levels.

However, the practical application of FS remains limited. Biological tissues contain numerous autofluorescent components—such as collagen and proteins—that generate significant background signals, making it difficult to isolate the relatively weak glucose signal. In addition, the photobleaching of fluorescent probes under prolonged excitation can reduce the signal stability, posing challenges for long-term monitoring [28].

Compared to NIR or MIR techniques, FS may offer superior sensitivity but requires more stringent control of the excitation conditions and sample composition.

##### Raman Spectroscopy

Raman spectroscopy (RS) relies on examining the frequency changes in scattered light. When light strikes a molecule, most of it undergoes Rayleigh scattering without any change in wavelength. However, a small portion of the light interacts with the molecule in a manner that results in Raman scattering, causing a shift in the wavelengths of the scattered light. These shifts are unique to the molecular composition of the material being investigated, allowing scientists to identify and analyze the characteristics of specific molecules by evaluating the properties of the Raman-scattered light.

In [29], the researchers used hemoglobin concentration as the standard and calculated the blood glucose concentration by analyzing the characteristic peaks of glucose and hemoglobin. They collected 25 Raman spectra from mice and obtained blood glucose reference values using a blood glucose meter. The final results indicated that the relationship between Raman intensity and glucose levels was nearly linear, with a correlation coefficient of 0.91, thereby confirming the feasibility of Raman spectroscopy for glucose detection.

While RS holds great potential, it is not without its challenges. A significant drawback is the inherently weak Raman signal, which is often several orders of magnitude less intense than Rayleigh scattering. This makes the technique particularly susceptible to background noise [30].

##### Optical Coherence Tomography

Optical Coherence Tomography (OCT) is an advanced imaging method that produces detailed, depth-specific visuals of biological tissues. This technique functions by merging light that bounces back from the tissue with light reflected from a reference arm within an interferometer, generating a coherent signal. Through the analysis of the resulting interference patterns, OCT delivers precise cross-sectional views of the tissue architecture. This capability has cemented its role as an indispensable tool in diagnosing conditions and tracking their progression [31].

In the context of non-invasive blood glucose monitoring, OCT has shown significant promise. For instance, Esenaliev R.O. and colleagues developed an OCT-based sensor. They tested the sensor using tissue phantoms and in vivo models, including pig and rabbit skin. After injecting glucose, OCT images were captured, and the results demonstrated a remarkable accuracy of 1% in measuring the scattering coefficient of the tissue. These findings indicate that OCT can effectively track changes in tissue scattering properties that correlate with variations in blood glucose levels [32].

Compared to other optical methods, OCT provides unparalleled spatial resolution and real-time imaging; however, it currently lacks the simplicity and miniaturization necessary for continuous or wearable applications. Future research should concentrate on enhancing the signal specificity, incorporating compact hardware platforms, and validating OCT-based glucose monitoring through human clinical trials.

Overall, optical sensor techniques have long been regarded as a promising foundation for non-invasive blood glucose monitoring due to their inherent sensitivity to molecular and structural changes in biological tissues. These approaches aim to capture the glucose-related variations in optical signals. However, many optical techniques still require bulky instrumentation, strict measurement conditions, and intensive computational modeling, limiting their translation into portable or wearable formats.

#### 2.2.2. Non-Optical Sensor Technologies

Non-optical sensor technologies include Bioimpedance Spectroscopy, Reverse Iontophoresis, Blood Substitute Measurement, Metabolic Balance, and Electromagnetic Techniques.

##### Bioimpedance Spectroscopy

Bioimpedance Spectroscopy (BIS) is a non-invasive technique used to evaluate the electrical properties of biological tissues in response to changes in blood glucose levels. By introducing a mild electrical current into the body, tissues exhibit both resistance and reactance. Since blood glucose levels affect the ionic concentration within tissue fluids, they subsequently alter the tissue’s conductivity and permittivity. This relationship enables the detection of blood glucose variations by analyzing changes in the body’s impedance [33].

Caduff et al. [34] developed a sensor technology based on BIS that is capable of analyzing the dielectric properties across a broad spectrum ranging from 1 to 200 MHz. They reported a strong correlation between high-frequency impedance signatures and venous blood glucose levels. The sensor boasts a resolution of 4 mg/dL, which is suitable for continuous glucose monitoring. However, performance variability significantly increased under real-life conditions, highlighting the sensitivity of BIS to confounding factors such as skin hydration, ambient temperature, and subject movement.

Despite its theoretical simplicity and low cost, BIS faces critical challenges in achieving the consistency and accuracy required for clinical use. Its readings are heavily influenced by individual anatomical differences (e.g., skin thickness, sweat glands) and extraneous physiological states unrelated to glucose, making robust calibration and compensation algorithms essential.

Nevertheless, BIS technology remains promising, particularly when integrated into multi-sensor platforms where it can complement other measurement methods. Its swift response, minimal energy demands, and compatibility with wearable devices make it appealing for incorporation into next-generation glucose monitoring systems.

##### Reverse Iontophoresis

Reverse Iontophoresis (RI) is a cutting-edge method that employs a gentle electric current to facilitate the movement of ions through the skin. By applying a low-level current, chloride ions are drawn toward the positive electrode, while sodium ions gravitate toward the negative one. This ion migration enables smaller molecules, such as water and glucose, to permeate the skin, whereas larger entities like proteins remain confined within the body [35].

A notable instance of RI is the GlucoWatch, developed by Cygnus [36]. This wearable device employs a low-level electric field on the skin, which prompts glucose particles in the interstitial fluid to migrate toward the surface. Upon reaching the skin, the glucose interacts with a gel layer integrated into the device, initiating a chemical reaction that enables glucose measurement. Despite its innovative design, the GlucoWatch faces challenges due to the limited strength of the electric current that it uses, which hinders the efficient movement of glucose molecules. Consequently, this can lead to inaccuracies and inconsistencies in readings, particularly in everyday situations where factors such as perspiration or skin imperfections can disrupt the measurement process.

##### Blood Substitute Measurement

Body fluids such as saliva, sweat, and urine offer a promising alternative to blood for the indirect monitoring of glucose levels. These fluids contain glucose levels that are closely correlated with those found in blood, facilitating the development of more convenient and less painful monitoring methods.

Zhang et al. [37] developed an integrated on-chip electrochemical biosensor capable of detecting glucose levels in saliva, demonstrating a significant correlation (R^2^ = 0.91) between salivary and blood glucose levels before and after oral glucose intake. The sensor achieved a detection limit of 1.2 μmol/L and showed potential for miniaturized, real-time saliva-based glucose monitoring. These findings support the feasibility of saliva as a diagnostic proxy for blood glucose under controlled conditions.

However, several obstacles must be addressed before these methods can become clinically viable. The composition of saliva can vary significantly between individuals and is influenced by factors such as hydration status, circadian rhythm, oral hygiene, and dietary intake. Moreover, glucose concentrations in saliva are typically 100–1000 times lower than in blood, necessitating highly sensitive detection techniques.

##### Metabolic Balance

Individuals with different glucose metabolism profiles exhibit significant variations in their resting energy expenditure, which can serve as an indicator of their blood glucose levels. Consider those with diabetes, for example. They often expend more energy at rest compared to those with stable glucose levels, primarily due to metabolic imbalances and the body’s difficulty in efficiently processing glucose. This correlation paves the way for innovative approaches to monitor glucose that incorporate various physiological data, such as temperature, humidity, blood oxygen levels, and heat changes, to indirectly assess blood glucose levels.

Based on this foundational concept, [38] introduced a non-invasive blood glucose monitoring technique that utilizes a metabolic heat conformation method. This innovative approach synthesizes information from multiple sensors to measure the essential physiological indicators, such as thermal output, blood circulation, and oxyhemoglobin levels. In experimental trials involving over 100 participants, the method achieved a correlation coefficient exceeding 0.85 when compared to invasive technologies, demonstrating its potential as a non-invasive sensing strategy.

Nonetheless, the effectiveness and reliability of this system hinge on the precision of the sensors and the robustness of the algorithm. Variables such as individual metabolic rate differences, the surrounding environmental factors, and sensor calibration can all influence the accuracy of the results.

##### Electromagnetic Techniques

A highly promising avenue in the realm of non-invasive glucose monitoring involves leveraging electromagnetic (EM) technology. As electromagnetic waves pass through human tissue, their fundamental properties, such as frequency, amplitude, and phase, are modulated by the tissue’s electrical and dielectric characteristics. These shifts provide critical insights into the tissue’s composition. EM-based methods have garnered considerable interest due to their affordability, straightforward fabrication processes, and adaptability in capturing diverse physiological signals. Numerous EM-driven approaches have been explored for glucose monitoring [39], highlighting their potential in this field.

Non-optical glucose sensing techniques encompass a variety of physical mechanisms, all aimed at overcoming the optical limitations of scattering, absorption overlap, and skin penetration depth by exploring alternative physiological indicators of glucose. However, this shift also introduces a new level of complexity; most of these methods measure indirect indicators rather than glucose directly, rendering them highly susceptible to confounding factors such as hydration, skin condition, and individual differences. Consequently, their effectiveness relies not only on the sensor hardware but also on the creation of reliable signal interpretation models that can differentiate glucose-specific signals from physiological noise. Thus, non-optical techniques present a promising yet inherently multivariate approach to non-invasive glucose monitoring.

In the subsequent sections, we will critically examine the most prominent EM-based glucose sensing methods, antenna-sensors, focusing on their underlying principles, design considerations, reported performance, and practical limitations in applications.

## 3. Antenna-Sensors

Antenna-sensors are a particularly promising avenue for non-invasive glucose monitoring, owing to their unique combination of sensing and wireless communication capabilities. Unlike traditional sensors, which frequently necessitate additional electronic components for signal transmission, antenna-sensors inherently serve as both detectors and data transmitters. This dual functionality allows for a simplified system architecture and passive operation, as sensing information can be encoded directly into the antenna’s signal without the requirement for external power sources. Moreover, antenna-sensors support multiplexing and distributed sensing, facilitating the creation of large-scale sensor arrays and multi-parameter platforms. Their structural versatility—including planar, low-profile, and flexible designs—enables seamless integration into wearable devices and curved body surfaces. Additionally, antenna-sensors can be produced at a low cost using established printed circuit board or additive manufacturing techniques. Their potential for engineering multi-modal sensitivity (e.g., dielectric, temperature, pressure) further broadens their applicability in personalized healthcare monitoring. For these reasons, antenna-sensors deserve focused attention as a highly relevant and versatile category of non-invasive biosensors within the context of next-generation glucose monitoring technologies.

Among its numerous critical radiation characteristics, the reflection coefficient (Γ) is pivotal in assessing the effectiveness of signal transfer. When the antenna’s input impedance at the feed point does not match precisely the feed line’s characteristic impedance, typically set at 50 ohms, the signal is not fully conveyed to the antenna. This impedance mismatch causes a portion of the signal to reflect back to the source, resulting in a measurable reflection coefficient [40].

The reflection coefficient indicates the proportion of the reflected signal’s amplitude to the incident signal’s amplitude, with both its magnitude and phase serving as indicators of impedance matching quality. When the reflection coefficient is near zero, it signifies excellent impedance matching, ensuring that the antenna effectively radiates the majority of the signal. Conversely, a reflection coefficient nearing one indicates a significant impedance mismatch, causing most of the signal to reflect back to the source rather than being transmitted. This coefficient can also be expressed using S parameters, particularly $S_{11}$, as detailed in Equation (1).

(1) $$S_{11}\left(dB\right)=20\log_{10}\left|\Gamma \right|$$

$S_{11}$ near 0 dB indicates a significant mismatch, with a large portion of the signal reflected. $S_{11}$ below −10 dB is generally considered good matching, as it means that less than 10% of the signal is reflected, allowing most of the energy to be radiated by the antenna.

**Figure 1:**
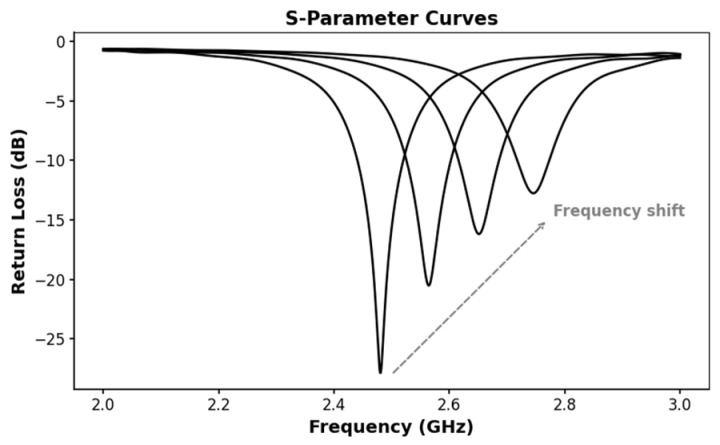
Illustration of frequencies shift of the antenna-sensor.

The resonant frequency is the specific point where $S_{11}$ reaches its lowest value, essentially marking the most efficient operating frequency. Bandwidth, on the other hand, defines the span of frequencies over which $S_{11}$ remains below −10 dB, indicating an optimal impedance match [41]. In theory, antenna-sensors can be used to measure a wide array of physical quantities by utilizing these metrics, including strain [42], temperature [43], and humidity [44], among others. For instance, when the physical quantities to be measured change, the antenna-sensor may experience a frequency shift, as depicted in Figure 1. This facilitates the conversion of the physical quantities into a measurable radiation parameter.

**Figure 2:**
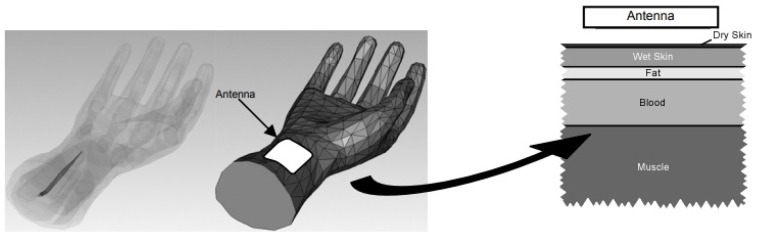
Human tissue phantom [46].

Antenna-sensors have also shown promise in monitoring blood glucose levels [45]. In a groundbreaking study by Feer [46], the viability of this method was first thoroughly explored. Feer’s experiments utilized a five-layer human tissue phantom (depicted in Figure 2) alongside a wideband monopole antenna-sensor positioned on the surface. The findings revealed that shifts in blood glucose levels altered the relative dielectric constant of the blood layer. These changes, in turn, had a direct impact on the antenna’s radiation properties, leading to detectable variations in its performance metrics. By capturing and analyzing these shifts, the antenna-sensor effectively correlated them with blood glucose concentrations, underscoring its potential as a non-invasive monitoring tool.

### 3.1. Rigid Antenna-Sensor

Traditional antenna-sensors are typically fabricated using rigid materials, which offer several notable advantages in their design and performance. First, rigid materials are strong and durable, making them highly resistant to external impacts, vibrations, and mechanical stress. This robustness ensures that the antenna-sensor maintains its structural integrity and functionality even in challenging environments. Second, rigid materials enable the fabrication of high-precision geometric shapes, which are critical for achieving the optimal radiation efficiency, precise impedance matching, and excellent directivity. These properties are essential for ensuring accurate and reliable signal transmission and reception in sensing applications. Additionally, rigid materials are widely available in the market and benefit from mature fabrication technologies, which streamline the manufacturing process and reduce the production costs. This cost-effectiveness makes rigid antenna-sensors an attractive option for large-scale deployment in various applications.

#### 3.1.1. Substrate Materials

Choosing the appropriate substrate is crucial in determining key performance metrics. Achieving the right balance among factors such as dielectric constant, loss tangent, thermal resilience, and mechanical integrity is essential to customize the substrate for specific scenarios. Table 2 showcases a variety of widely used rigid substrates in antenna-sensor development, each with a unique set of characteristics that make them suitable for different applications [47].

#### 3.1.2. Conductive Materials

Conductive materials act as the primary medium for signal transmission and reception. Therefore, the choice of conductive material is critical in antenna-sensor design, as it significantly affects the efficiency, conductivity, and overall performance of the antenna-sensor. The key considerations include electrical conductivity, oxidation resistance, thermal stability, and mechanical durability to ensure reliable operation under varying environmental conditions. Table 3 shows some commonly used rigid conductive materials in antenna-sensor design [48].

#### 3.1.3. Fabrication Methods

Antenna fabrication methods vary depending on the material properties, application requirements, and performance considerations, with PCB etching and mechanical machining being two commonly used techniques.

The PCB etching process is a widely used method for fabricating rigid patch antennas. It employs photolithography and chemical etching to selectively remove excess copper from a dielectric substrate, forming the desired antenna pattern with high precision. Due to its cost-effectiveness, scalability, and compatibility with high-frequency applications, PCB etching has become the dominant technique for fabricating RF and microwave antennas.

The mechanical machining process is primarily used for fabricating rigid metal antennas, including parabolic reflectors, waveguide antennas, and high-gain structures. This method includes CNC machining, laser cutting, and metal casting, enabling the production of highly conductive, low-loss antennas with superior mechanical durability. Compared to PCB etching, mechanical machining is better suited for high-power applications and extreme environments, such as satellite communications and radar systems, where robustness and performance reliability are critical.

#### 3.1.4. Applications

Currently, there have been considerable attempts to use rigid antenna-sensors for non-invasive blood glucose monitoring. Various types of antenna-sensors have demonstrated their significant potential in this field.

##### Patch Antenna-Sensors

The patch antenna is distinguished as one of the most essential and frequently used types of antennas, particularly in modern wireless communication systems. It typically consists of a metallic patch affixed to a dielectric substrate, with a ground plane positioned on the opposite side.

**Figure 3:**
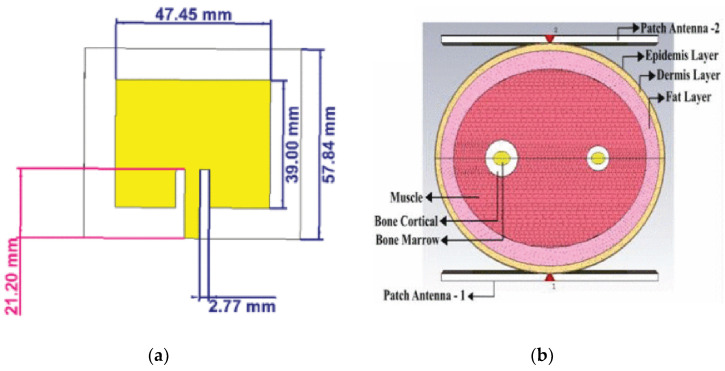
(**a**) Design of the patch antenna-sensor. (**b**) Forearm phantom placed between two patch antenna-sensors [49].

The researchers in [49] developed a patch antenna-sensor (depicted in Figure 3a) fabricated on a Rogers RT5870 substrate. This innovative design exhibited remarkable performance, boasting a reflection coefficient of −48.28 dB and an impressive gain of 7.004 dBi. To assess its potential for non-invasive blood glucose monitoring, simulations were conducted using an elliptical cylindrical model of a human forearm, measuring 70 mm along the major axis and 50 mm along the minor axis. The forearm model was placed between two patch antenna-sensors, as illustrated in Figure 3b, to replicate practical monitoring scenarios. The findings indicated a strong correlation between shifts in the blood’s dielectric constant and fluctuations in the $S_{21}$ parameter. Notably, a 20-unit change in the dielectric constant of blood resulted in an approximate 0.64 dB variation in the $S_{21}$ parameter, highlighting the antenna-sensor’s capability to detect glucose-related changes in blood characteristics.

**Figure 4:**
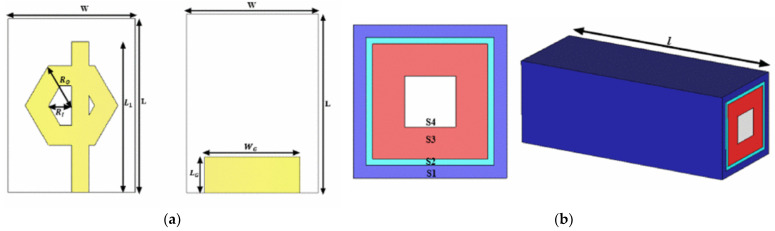
(**a**) Proposed ϕ-shaped antenna-sensor. (**b**) Four-layer finger phantom [50].

Similarly, reference [50] proposed a planar ϕ-shaped antenna designed specifically for fingertip measurements, as illustrated in Figure 4a. The study utilized a four-layer finger phantom consisting of skin, fat, muscle, and blood-equivalent layers (Figure 4b) to evaluate frequency shifts corresponding to glucose variations. When the simulated blood glucose level varied from 0 to 1000 mg/dL, the antenna’s maximum resonant frequency reached 75 MHz, demonstrating the system’s capability to respond to a broad physiological blood glucose range.

##### Metamaterial Antenna-Sensors

Antenna-sensors inspired by metamaterials offer novel possibilities for non-invasive glucose monitoring by leveraging the unique electromagnetic properties of artificially engineered structures. Features such as negative permittivity, bandgap suppression, and high field confinement enhance the sensitivity to dielectric changes in biological media. Common building blocks include complementary split-ring resonators (CSRRs), electromagnetic bandgap (EBG) structures, and Jerusalem Cross (JC) resonators, which all enable tight spatial energy localization and sharp frequency responses.

**Figure 5:**
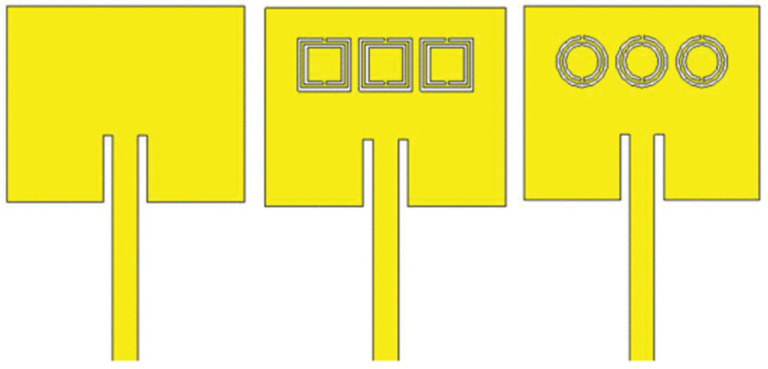
Antenna-sensors with different shaped CSRRs [51].

In [51], the authors investigated planar antenna-sensors incorporating complementary split-ring resonator (CSRR) structures for detecting glucose concentrations in aqueous solutions. Two geometrical configurations—square and circular CSRRs—were evaluated in terms of their sensitivity, measured via changes in the reflection coefficient across varying glucose levels, as shown in Figure 5. To quantify the relationship between the glucose concentration and antenna response, a mathematical model was developed linking the concentration levels to the reflection parameter. The experimental results demonstrated that the circular CSRR exhibited superior sensitivity, achieving a shift of 4.37 dB per mg/mL, compared to 1.16 dB per mg/mL for the square configuration. This enhancement was attributed to improved electric field confinement and the enhanced resonant stability provided by the circular geometry’s symmetrical structure.

**Figure 6:**
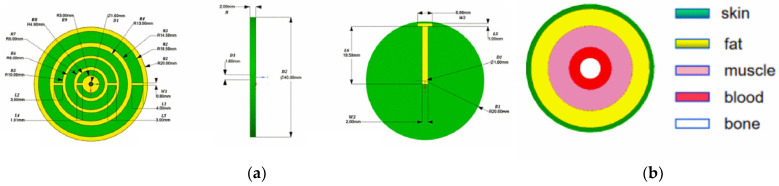
(**a**) Three-view of antenna-sensor. (**b**) Forearm tissue phantom [52].

Built upon this concept, [52] introduced a multi-band antenna-sensor featuring a four-ring CSRR configuration, tailored for glucose sensing specific to tissue layers, as shown in Figure 6a. A forearm phantom, consisting of skin, fat, muscle, blood, and bone layers, was created to assess the penetration of electromagnetic waves and the return loss performance, as depicted in Figure 6b. The antenna exhibited a peak sensitivity of 1.6 × 10⁻³ dB/(mg/dL) across the frequency bands, showcasing the potential of multi-resonance designs to distinguish glucose-induced permittivity changes at different depths. Furthermore, the study incorporated a gradient boosting decision tree (GBDT) machine learning algorithm to correlate antenna outputs with glucose concentrations. The model achieved a coefficient of determination (R²) exceeding 98%, signifying robust predictive power.

These studies demonstrate the advantages of metamaterial structures in enhancing glucose detection resolution through sharp resonances, spatial selectivity, and multi-band operation. Their compactness and sensitivity to micro-level dielectric changes make them promising candidates for next-generation rigid sensor platforms. Further research is required to evaluate their long-term stability, manufacturability, and real-world adaptability in wearable, non-invasive systems.

##### UWB Antenna-Sensors

Ultra-wideband (UWB) antennas are characterized by their exceptionally wide operating frequency range, exceeding 500 MHz. UWB communication systems operate across a broad frequency spectrum, typically from 3.1 GHz to 10.6 GHz, as stipulated by FCC regulations. These systems often employ various structures, including planar monopole, Vivaldi, or bowtie designs. The defining feature of UWB antennas is their ability to transmit and receive signals over an extensive bandwidth. This broad spectrum is attainable through specific antenna geometries and broadband feeding techniques, making them ideal for high data rate communications, radar, and medical sensing applications.

**Figure 7:**
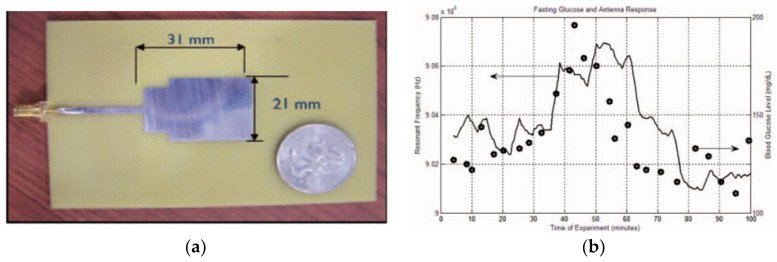
(**a**) Proposed UWB antenna-sensor. (**b**) Monitoring of resonant frequency and blood glucose levels [53].

In [53], the researchers developed a compact UWB antenna-sensor (depicted in Figure 7a) by integrating a rectangular matching segment into the radiating patch, resulting in extended bandwidth coverage between 1 and 6 GHz. This design enabled the antenna to interact with a wider range of tissue-layer resonances. An in vivo experiment was conducted in which the antenna was affixed to a subject’s forearm while glucose intake was administered. The sensor’s resonant frequency exhibited a positive correlation with the blood glucose concentration. As the glucose levels climbed, the sensor’s resonant frequency followed suit, as shown in Figure 7b. This confirmed the sensor’s capability to track physiological changes in real time by monitoring the dielectric response variations in the skin and subcutaneous layers. Their next goal is to develop a mathematical model that correlates the antenna response with blood glucose levels.

**Figure 8:**
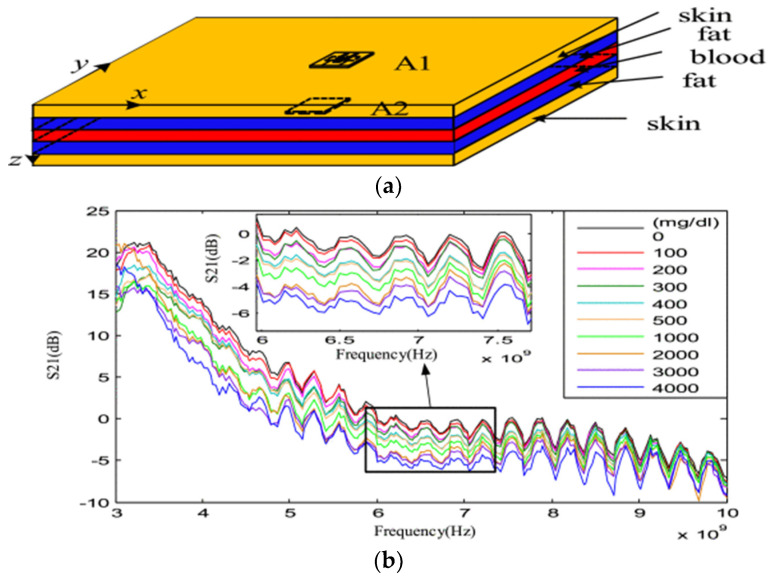
(**a**) Earlobe phantom (A1: Antenna 1, A2: Antenna 2). (**b**) $S_{21}$ of the detected signals of the range of the blood glucose levels [54].

In a separate study [54], a novel UWB antenna-sensor was introduced, featuring a fork-shaped microstrip feedline designed to achieve enhanced impedance matching across a broad operating range. To assess its efficacy, the team employed an earlobe phantom (depicted in Figure 8a) that mimicked the dielectric properties of human tissue. The setup involved positioning UWB antenna-sensors on either side of the phantom, with a network analyzer capturing the necessary data. By monitoring the parameter, they tracked alterations in the signal’s energy as the glucose levels changed. The findings highlighted a distinct pattern at 6.5 GHz: as the glucose levels rose, the signal’s peak energy density gradually declined, as visualized in Figure 8b. This inverse correlation highlights the antenna-sensor’s precision in detecting glucose-induced changes in tissue properties. Nevertheless, the experimental model’s architecture, the input microwave frequency range, the antenna design, and the specific algorithm all necessitate meticulous planning to achieve optimal correspondence.

##### Resonator Antenna-Sensors

Dielectric resonator antennas and patch antennas share numerous similarities. Both are miniaturized antennas that function similarly to resonant cavities and can calculate the radiation field by determining the surface equivalent magnetic current. The primary difference lies in their radiation mechanisms: patch antennas emit radiation through the gaps between conductors, whereas dielectric resonator antennas radiate across their entire outer surface, excluding the ground plane.

**Figure 9:**
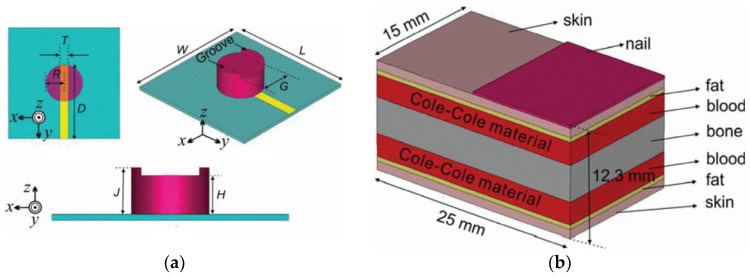
(**a**) Proposed antenna-sensor. (**b**) Thumb tissue phantom in CST microwave studio [55].

In a study [55], the researchers introduced a cylindrical resonator antenna-sensor (Figure 9a) tailored for non-invasive blood glucose monitoring. The device functions by having the patient place their thumb on it, enabling the detection of glucose level changes through shifts in the dielectric characteristics of blood. To simulate the antenna-sensor’s performance in a real-world setting, the team utilized a human thumb phantom in CST software (Figure 9b). This allowed for a detailed examination of the sensor’s interaction with the human body. To bolster the simulation’s precision, the researchers incorporated the Cole–Cole model, a widely recognized approach for modeling the complex dielectric constant of biological tissues. The study’s findings revealed a distinct correlation between the antenna-sensor’s resonant frequency and alterations in the blood’s dielectric constant, which are influenced by glucose level fluctuations. As the glucose levels changed, the blood’s dielectric properties shifted, resulting in a corresponding adjustment in the antenna-sensor’s resonant frequency. The sensitivity of the proposed sensor can reach 2.81 kHz/mg/dL.

**Figure 10:**
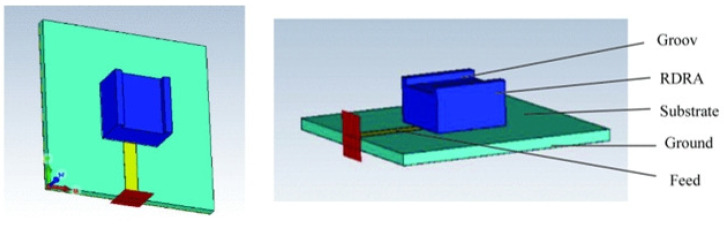
Proposed antenna-sensor [56].

In [56], the researchers unveiled a novel rectangular dielectric resonator antenna-sensor (depicted in Figure 10) engineered to function at 7.19 GHz. This device was tailored for non-invasive blood glucose monitoring, leveraging the distinctive characteristics of dielectric resonators to deliver high sensitivity and precision. To assess its efficacy, the team used a human thumb phantom in CST (Figure 9b), positioning it directly on the sensor to mimic real-world glucose detection scenarios. Upon contact with the thumb phantom, the antenna-sensor’s resonant frequency dropped. As the glucose level in the phantom increased from 0 mg/dL to 2000 mg/dL, the resonant frequency further decreased from 5.234 GHz to 5.204 GHz. These results underscore the antenna-sensor’s ability to monitor blood glucose changes by tracking the shifts in resonant frequency, showcasing its potential for real-time, non-invasive glucose monitoring applications.

In summary, a variety of rigid antenna-sensor configurations have been proposed and studied for non-invasive glucose monitoring, each offering distinct advantages in terms of sensitivity, design flexibility, and modeling potential. Patch antenna-sensors remain the most widely adopted due to their compact structure, high radiation efficiency, and well-understood resonant behavior, enabling the reliable detection of glucose-induced dielectric shifts in tissue phantoms. Metamaterial antenna-sensors demonstrate enhanced field confinement and ultra-sharp resonances, which translate into higher detection sensitivity and more compact designs. Meanwhile, UWB antenna-sensors provide broadband coupling to layered biological structures and allow frequency-specific penetration depth analysis, supporting dynamic, real-time glucose tracking under practical conditions. Additionally, dielectric resonator antenna-sensors have shown great promise for their low-loss performance, high Q-factor, and ability to interact volumetrically with tissue, offering stable frequency shifts even in compact contact configurations such as fingertip sensing.

These studies collectively underscore the adaptability and increasing sophistication of rigid antenna-sensor technologies within the field of electromagnetic glucose sensing. Although contemporary research primarily depends on simulations and phantom-based assessments, the established sensitivity to glucose-induced dielectric changes lays a solid groundwork for the progression to experimental prototyping, hardware incorporation, and ultimate clinical application. Nevertheless, obstacles such as bulkiness, insufficient flexibility, and user discomfort continue to impede their appropriateness for prolonged wearable application—issues that are progressively being tackled by the advent of flexible antenna-sensor platforms, as explored in the subsequent section.

### 3.2. Flexible Antenna-Sensors

Flexible electronics have emerged as a groundbreaking innovation within Body Area Networks (BANs) [57], significantly impacting areas such as healthcare, sports, and virtual reality. Unlike conventional rigid electronics, these flexible alternatives are designed to be lightweight, adaptable, and easily conformable, making them ideal for integration into wearable technologies and other systems that interface closely with the human body. This flexibility effectively addresses the limitations of traditional rigid electronics.

Flexible electronics, particularly flexible antenna-sensors, are of paramount importance. These antennas are engineered to offer exceptional mechanical flexibility, enabling them to adapt to non-flat surfaces, such as the human body, without sacrificing performance. This flexibility guarantees that the antenna-sensors retain robust wireless communication and sensing capabilities, even when bent, stretched, or twisted.

#### 3.2.1. Substrate Materials

For flexible antenna-sensors, the ideal substrate materials exhibit minimal thermal expansion, low relative permittivity, and superior thermal conductivity [58]. A variety of materials have shown considerable promise in this capacity, as detailed in Table 4.

Kapton has established itself as a preferred substrate in the realm of flexible electronics, celebrated for its outstanding structural integrity and dielectric stability, even when subjected to mechanical stress. These remarkable characteristics position Kapton as an outstanding option for projects that demand toughness and dependability in ever-changing environments [59]. In [60], the authors introduced a wideband antenna based on Kapton, specifically tailored for flexible millimeter-wave applications. This antenna boasts an impressive bandwidth of 57.3%, making it particularly well-suited for flexible 5G wireless technologies. The design of this antenna ensures that it maintains its wideband performance, even under bending conditions, a vital attribute for wearable devices and various other flexible electronic applications.

Polyethylene terephthalate (PET) is a widely used thermoplastic polymer resin, primarily employed in the production of synthetic fibers [61]. It is lightweight and thin, making it ideal for integration into antenna designs. For instance, in [62], the researchers designed a planar inverted-F antenna (PIFA) using PET as the substrate, targeting car applications. They selected PET because it maintains its performance whether flat or bent into a curve. The study demonstrated that the antenna’s gain remained virtually unchanged in both scenarios, confirming that PET is a dependable and resilient substrate for antennas requiring flexibility.

Polydimethylsiloxane (PDMS) is an adaptable material that is valued for its chemical resistance, high-temperature tolerance, and gas permeability, as well as its uniform and consistent properties. These characteristics make PDMS an ideal substrate for creating antennas that are flexible and stretchable [63]. A development detailed in [64] revealed that the researchers had created a dual-band flexible antenna based on a 2 mm PDMS substrate. This innovative antenna is engineered to provide optimal performance across two separate frequency ranges, thereby extending its utility in various wireless communication applications. A significant benefit of this antenna is its minimal distortion from bending, ensuring reliable operation even when deformed. This feature is crucial for its integration into flexible electronic systems, where maintaining operational integrity on irregular or moving surfaces is essential.

Liquid Crystal Polymer (LCP) is a top-tier thermoplastic, celebrated for its remarkable ability to resist heat, chemicals, and moisture. These standout features, combined with its minimal dielectric loss, superb dimensional stability, and resilience in tough conditions, position LCP as an excellent material for millimeter-wave systems and cutting-edge wireless technologies [65]. Its unwavering performance in extreme scenarios makes it a trusted choice in high-stakes industries such as aerospace, automotive, and industrial applications. In [66], the researchers developed a multi-band antenna for the ISM band, utilizing an LCP substrate. By leveraging LCP’s distinctive characteristics, the antenna delivers consistent and efficient operation across various frequency ranges.

**Table 4:** Commonly used flexible substrate material in antenna-sensor design.

Reference	Substrates	Dielectric Constant	Dielectric Loss	Thickness
[60]	Kapton	3.5	0.002	100 μm
[62]	PET	2.63	0.004	0.5 mm
[64]	PDMS	2.65	0.02	2 mm
[66]	LCP	3.16	0.025	4 mm
[67]	Paper	1.9	0.025	100 μm

Note: The materials listed in this table represent commonly used examples in the literature and do not constitute an exhaustive list of all available material options.

Paper substrates represent a significant and eco-friendly advancement in the field of flexible electronics, combining adaptability, a slim profile, and the ability to seamlessly integrate into compact, portable devices [68]. They are not only cost-effective and suitable for large-scale production but also stand out as a preferred choice for environmentally conscious applications due to their green attributes. Their lightweight nature and biodegradable properties further establish them as an excellent choice for wearable devices that are in direct contact with the skin. In [67], the researchers presented a flexible antenna-sensor made from paper, specifically designed for biosensing applications. This innovative device leverages the inherent properties of paper substrates, such as their ability to absorb liquids and their straightforward manufacturing processes, to provide an affordable and versatile sensing solution. The porous structure of paper facilitates the interaction with bodily fluids, enabling the detection of biomarkers or changes in physiological states, such as variations in glucose levels.

In flexible glucose monitoring applications, the choice of substrate must consider not only the electromagnetic performance but also the mechanical compliance, biocompatibility, and fabrication feasibility. For instance, PET and PDMS are well-suited for wearable, skin-contact devices due to their flexibility and comfort, while materials such as Kapton and LCP provide greater stability for high-frequency and multi-band designs. Paper substrates, although less durable, are advantageous in disposable or low-cost biosensing contexts. Ultimately, the material selection should be guided by the specific application scenario—whether for continuous monitoring, short-term use, or integration into textiles or epidermal platforms.

#### 3.2.2. Conductive Materials

For flexible antenna-sensors, achieving conductive patterns with high conductivity is crucial to ensure high gain, efficiency, and bandwidth. Adhesive copper [69], copper tapes [70], and copper cladding [71] are widely used materials for flexible antenna design, offering exceptional electrical conductivity and mechanical flexibility. These materials are easy to use, conform well to complex curved surfaces, and are ideal for the rapid prototyping of antenna setups. However, they may suffer from adhesive degradation and mechanical wear over time, which could affect their performance.

Nanoparticle inks, especially those based on silver or copper, have become essential for flexible antenna-sensors. Silver nanoparticle inks exhibit excellent conductivity and oxidation resistance; however, their high cost restricts their use in cost-sensitive applications. In contrast, copper nanoparticle inks provide a more cost-effective alternative with good conductivity. Nevertheless, copper’s susceptibility to oxidation may degrade performance, necessitating additional surface protective measures. Despite these differences, both silver and copper nanoparticle inks enable precise, scalable, and versatile antenna designs through methods such as inkjet printing [72].

Conductive polymers, such as polyaniline (PANI) [73] and polypyrrole (PPy) [74], are appealing for use in flexible antenna-sensors due to their adaptability, reconfigurability, and mechanical flexibility. Compared to PPy, PANI exhibits greater chemical stability, rendering it more appropriate for specific applications. Nevertheless, its relatively low electrical conductivity poses a limitation. To overcome this issue, researchers have suggested incorporating carbon nanotubes into the polymer matrix to improve its conductivity [75,76]. The aforementioned materials are summarized in Table 5 for comparison purposes.

Ultimately, the optimal choice hinges on the intended application scenario—whether it prioritizes high gain, stretchability, low cost, or integration into emerging printing technologies. Hybrid approaches that combine multiple materials may offer a promising direction to meet the diverse functional and performance requirements.

#### 3.2.3. Textile Antenna-Sensors

Despite their advantages, the aforementioned flexible materials have limitations in terms of comfort, breathability, and seamless integration with everyday clothing. Consequently, textile antenna-sensors are attracting more attention. The most common method to realize textile antenna-sensors involves substituting the substrate and conductive elements of traditional antennas with textile materials, such as using felt for the substrate and conductive textiles for the conductive layers.

The dielectric properties of textiles have been thoroughly examined in [77]. Various methods have been employed to evaluate these features, including the cavity perturbation technique [78], the Method of Moments segment approach [79], the free-space technique [80], and the transmission line method [81]. Due to their porous nature, textiles generally exhibit a lower relative permittivity compared to conventional rigid substrates. Table 6 provides a summary of the dielectric characteristics of widely used commercial textiles.

Furthermore, the moisture absorption capacity of textile fabrics significantly affects their dielectric behavior. Since water has a higher dielectric constant, its absorption into the textiles modifies the material’s electromagnetic properties, resulting in an increase in both the overall dielectric constant and dielectric loss [83].

To optimize the efficiency of textile antenna-sensors, the conductive materials used must have low resistivity [84]. In addition to commonly utilized nanoparticle inks, e-textile materials such as Zelt, Pure Copper Taffeta Fabric, and Shieldit Super (Table 7) are frequently employed in the design of textile antennas. Maintaining the breathability, flexibility, appearance, and washability of textiles after electronic integration are major challenges for electronic textile applications.

#### 3.2.4. Fabrication Methods

##### Screen Printing

**Figure 11:**
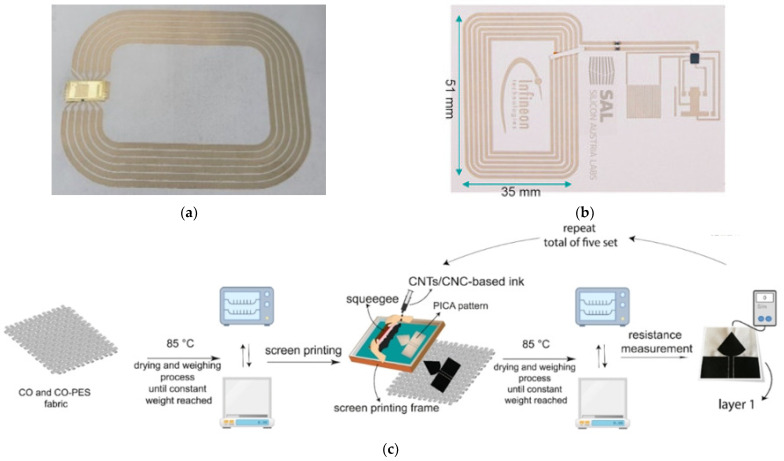
(**a**) Screen printed RFID antenna-sensor [86]. (**b**) Screen-printed antenna-sensor prototype on paper substrate [87]. (**c**) Schematics of screen printing of antenna-sensors [88].

Screen printing is an efficient, rapid, and economical technique for fabricating flexible electronics. It involves pushing conductive ink through a screen with a predefined pattern onto a substrate, enabling the creation of precise antenna structures [85]. This method facilitates large-scale production while maintaining good electrical performance. Figure 11 presents examples of antenna-sensors fabricated using screen-printing techniques, demonstrating their potential for flexible and wearable applications.

Despite these benefits, screen printing has certain limitations, such as restrictions on the number of achievable layers, imprecise control over the thickness of conductive layers, and relatively low printing resolution [89,90]. These drawbacks necessitate further advances in printing technology and material formulation.

##### Inkjet Printing

Inkjet printing is recognized for its ability to produce highly accurate patterns. It achieves this by depositing ink droplets as small as a few picoliters with remarkable precision. By carefully placing individual droplets, this method reduces the material waste and offers an economical alternative to traditional etching processes [91].

**Figure 12:**
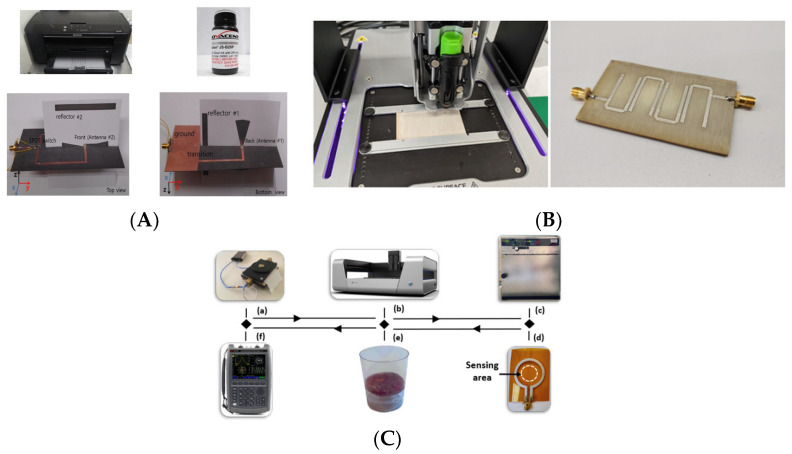
(**A**) Figures of home inkjet printer, silver nanoparticle ink, and fabricated antenna-sensor [93]. (**B**) V-One inkjet printer and inkjet-printed antenna-sensor [94]. (**C**) Experimental setup and fabrication process of antenna-sensor for monitoring dielectric changes in meat (a) Q-meter used for extracting electrical properties of the substrate; (b) Voltera NOVA inkjet and extrusion printer used for antenna fabrication; (c) Memmert oven for drying and curing the printed ink to ensure proper adhesion and distribution; (d) final fabricated loop antenna; (e) 10 g of meat sample; (f) vector network analyzer (VNA) for antenna performance validation [95].

However, inkjet printing technology faces challenges, including the limited compatibility of certain conductive inks, potential degradation of conductivity over time, and susceptibility to mechanical stress [92]. Figure 12 presents examples of antenna-sensors fabricated using inkjet printing.

##### Embroidering

The embroidery method is commonly used in the fabrication of textile antenna-sensors. Unlike other techniques, it does not rely on adhesive materials that could compromise the fabric’s electrical properties. Instead, this approach utilizes an automated embroidery machine to sew conductive thread directly onto a textile substrate. The process begins with designing the antenna layout, which is then converted into a stitch pattern using specialized software. The conductive thread is precisely sewn according to the design, forming the antenna’s radiating element [96].

**Figure 13:**
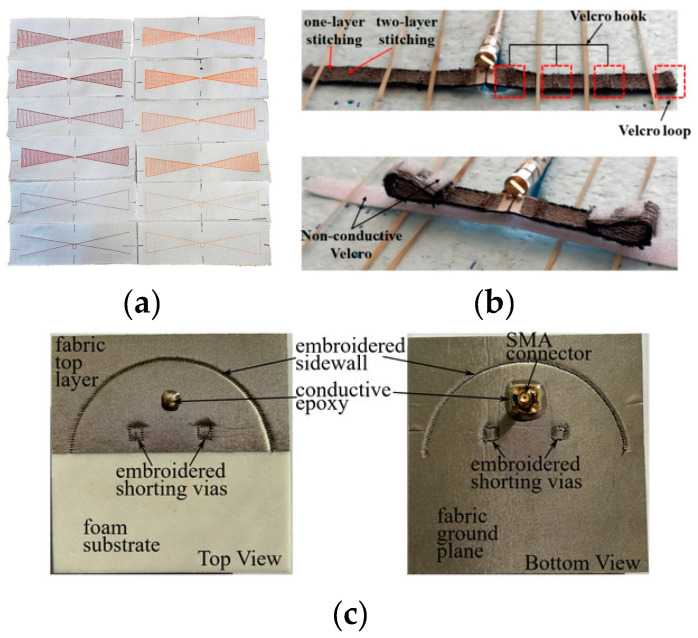
(**a**) Embroidery bow-tie antenna-sensor prototype [98]. (**b**) Flat prototype and folded prototype of embroidery antenna-sensor [99]. (**c**) Embroidery antenna-sensor prototype [100].

Embroidery antenna-sensors have certain limitations when compared to metal antenna-sensors. For instance, conductive yarns have high stretchability and low resolution, making it challenging to achieve fine geometries. Additionally, most conductive yarns are made with a nylon core coated in silver or carbon, materials that possess significantly higher resistivity compared to metal antennas [97]. Figure 13 shows some examples of antenna-sensors fabricated using the embroidery technique.

##### Weaving

**Figure 14:**
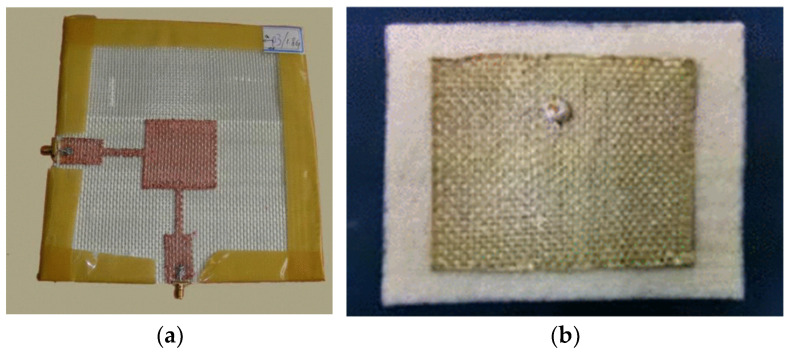
(**a**) Proposed woven antenna-sensor [101]. (**b**) Woven antenna-sensor on a felt substrate [102].

Weaving is another common method for fabricating textile antenna-sensors. In this process, conductive threads are interlaced with non-conductive threads in a systematic pattern, resulting in a uniform and durable structure. This approach allows for precise control over the placement and density of the conductive elements, enabling the creation of antennas with consistent electrical properties. However, weaving has its limitations, such as reduced design flexibility, and it may require more conductive material to achieve the desired performance. Figure 14 illustrates some antenna-sensors fabricated using the weaving method.

In the context of non-invasive glucose monitoring, the selection of the fabrication method for flexible antennas is crucial in determining the sensing performance, mechanical durability, and integration with the human body. Screen printing is advantageous due to its cost-effectiveness and high material deposition efficiency, making it suitable for mass production. However, its reliance on stencils limits design flexibility and makes it less ideal for rapidly iterating glucose sensor prototypes. Inkjet printing, on the other hand, allows for precise, maskless deposition of conductive inks and is well-suited for fabricating complex and miniaturized antenna geometries required for high-frequency glucose sensing. Its drawbacks include its sensitivity to the environmental conditions and slower throughput, which may limit its large-scale deployment.

Embroidery provides excellent mechanical stability and strong adhesion to textile substrates, which is advantageous for daily wear and long-term glucose monitoring. However, its relatively low resolution and increased surface roughness may compromise its electromagnetic performance. Weaving allows antennas to be seamlessly integrated into clothing during the textile-manufacturing stage, offering superior comfort and durability. Nevertheless, it provides the least design freedom and lowest precision compared to the other three methods.

In summary, for high-precision sensing applications such as continuous glucose monitoring, inkjet printing is typically preferred due to its accuracy and adaptability. For robust, long-term wearability, embroidering or weaving may be more appropriate. Screen printing excels in scalable production when the design is already optimized. Therefore, the choice of fabrication method should be application-driven, balancing resolution, flexibility, stability, and cost.

#### 3.2.5. Human Impact

The effectiveness of antenna-sensors initially designed for use in free space can be significantly affected when placed near the human body. This impact primarily arises from the high dielectric constant and loss properties of human tissues, which can alter the electromagnetic environment. As a result, these changes lead to an impedance mismatch and a shift in resonance frequency. Additionally, the substantial coupling between the antenna-sensor and the human body increases the absorption of electromagnetic energy, reduces the radiation efficiency, and may raise the specific absorption rate (SAR), thereby posing potential safety concerns.

Human tissue consists of a variety of intricate, non-uniform organic substances, each with unique electromagnetic properties. In biomedical applications, two primary models are predominantly used for tissue representation: the Debye model [103] and the Cole–Cole model [104]. The Cole–Cole model is generally preferred for its superior accuracy in depicting the dielectric behavior of tissues. According to this model, the complex permittivity of blood can be characterized via Equation (2). Utilizing the Cole–Cole framework, human tissue phantoms, such as those resembling fingers or forearms, can be constructed. In this method, only the blood layer is modeled as a Cole–Cole material, while other layers, including bone, muscle, fat, and skin, are treated as uniform dielectric materials with constant dielectric properties.

(2) $$\hat{\varepsilon }=\varepsilon_{\infty }+\sum_{n}\frac{∆\varepsilon_{n}}{1+{\left(jw\tau_{n}\right)}^{1-\alpha_{n}}}+\frac{\sigma_{i}}{jw\varepsilon_{0}}$$

##### Phantom

**Figure 15:**
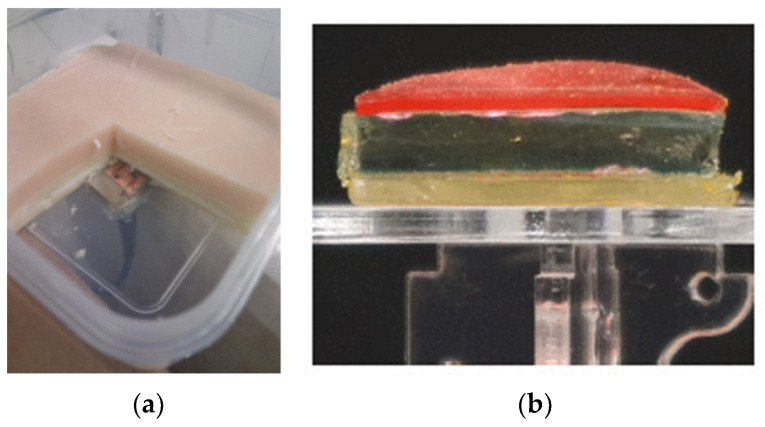
(**a**) Four-layered phantom placed over the sensor [105]. (**b**) Three-layer body phantom [106].

To effectively replicate the interaction between an antenna and the human body, it is crucial to construct human phantoms with precision. Researchers have explored a range of techniques, as documented in academic studies. For instance, one approach outlined in [105] involves crafting semisolid oil-in-gelatine dispersion phantoms from readily available materials (as illustrated in Figure 15a). This method relies on fine-tuning the dielectric properties by adjusting the mixture: water increases the relative dielectric constant, sodium chloride enhances the conductivity, oil decreases both the relative permittivity and conductivity, and gelatine acts as the binding agent to solidify the structure.

In [106], a multilayer phantom (Figure 15b) was designed using polymers capable of absorbing different complex liquids, as described in [107]. Different polymers were used for each layer: for example, polyacrylamide swollen in a 15% acetonitrile aqueous mixture was used for muscle, while polyhydroxyethyl acrylate swollen with ethanol was used for fat. Since the polymers expand during synthesis, they were prepared within 3D-printed glass molds with controlled gaps to achieve the desired layer thicknesses.

##### SAR

The SAR (Specific Absorption Rate) measures the amount of electromagnetic energy absorbed by the human body and is primarily used to assess the potential health effects of wireless technology. The European Union has established stringent SAR limits to protect individuals from potential health risks. According to EU regulations, the maximum permissible SAR for devices in contact with the body is set at 2.0 W/kg, averaged over 10 g of tissue, for localized exposure to the head and torso. For devices worn on the limbs, the limit can be as high as 4.0 W/kg. China has adopted SAR standards that are identical to those of the EU. In contrast, the United States has set a SAR limit of 1.6 W/kg for the head and torso, without a distinct limit for limb exposure [108]. These regulations are designed to reduce the thermal effects caused by the absorption of radio frequency energy.

##### Blood Glucose Measurement Regulation

The European Union adheres to the ISO 15197:2015 standard [109], which establishes stringent precision benchmarks for blood glucose monitoring devices. Within this framework, when blood glucose levels are below 5.55 mmol/L, 95% of readings must fall within ±0.83 mmol/L of the lab-based reference. Conversely, for levels at or above 5.55 mmol/L, 95% of results should remain within ±15% of the reference value. These rigorous criteria ensure the clinical reliability, safety, and accuracy of glucose monitoring systems, ultimately supporting optimal patient care and management.

#### 3.2.6. Data Processing and Modeling

Unlike sensors that respond directly to glucose molecules, antenna-sensors monitor blood glucose levels by detecting minute perturbations in the electromagnetic properties of multilayer biological tissues. These perturbations alter electromagnetic wave behavior, such as shifts in resonant frequency or alterations in bandwidth. However, such changes are often subtle, nonlinear, and entangled with a variety of confounding factors.

While the forward relationship—glucose affecting tissue permittivity—is reasonably modeled using empirical dielectric dispersion frameworks such as the Cole–Cole equation, the inverse problem of estimating blood glucose levels from observed antenna responses is controversial. Several structural challenges characterize this problem:

1. Non-uniqueness: Distinct dielectric distributions can produce similar antenna responses.
2. Instability: Minor variations in skin–sensor coupling, motion, or environmental humidity may induce disproportionate shifts in signal.
3. High dimensionality: Measured features span multiple domains (e.g., frequency, amplitude, phase), yet the features that are truly related to glucose levels are limited.

These characteristics necessitate the application of advanced signal processing and modeling techniques to extract relevant features and stabilize predictive models. In the preprocessing stage, methods such as wavelet packet decomposition, empirical mode decomposition, and spectral filtering are employed to enhance the signal-to-noise ratio, isolate local resonant shifts, and suppress spurious background effects.

On the modeling side, a range of supervised ML approaches have been explored to learn the nonlinear mapping between processed features and glucose concentration. Notable among them are the following:

1. Gradient Boosting Decision Trees: Effective for tabular feature sets, capable of modeling complex interactions while maintaining interpretability.
2. Support Vector Machines: Well-suited for high-dimensional feature spaces with limited training data.
3. Artificial Neural Networks and deep convolutional models: Particularly useful when raw spectral inputs are available, though requiring substantial data volume and careful regularization.

Despite their promise, these methods face critical limitations. Most available datasets are derived from phantom experiments or simulations, limiting biological variability and real-world realism. Inter-individual physiological variability—including differences in skin thickness, hydration, and vascular structure—introduces domain shifts that degrade model generalization. Real-time deployment also imposes constraints on computational latency, power consumption, and adaptive calibration under dynamically changing contact and environmental conditions.

Ultimately, antenna-based glucose estimation is not merely a regression problem but a case of robust inverse modeling under multi-factorial uncertainty. Progress in this area depends on the convergence of hardware innovation, dielectric modeling, and machine learning to jointly address the complexity, variability, and real-time requirements of practical, wearable glucose sensing systems.

#### 3.2.7. Blood Glucose Monitoring Application

**Figure 16:**
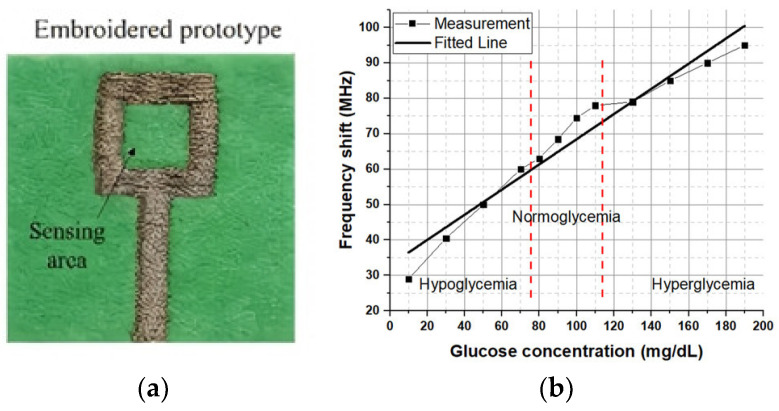
(**a**) Embroidery process of the proposed antenna-sensor. (**b**) Linear correlation for the frequency shift as a function of glucose concentration in different diabetic conditions [110].

El Gharbi et al. proposed a textile-based monopole antenna-sensor designed for the in vitro monitoring of blood glucose levels. The device was fabricated using embroidery techniques, where a silver-plated conductive yarn was stitched onto a non-woven polyester felt substrate to create a square-ring monopole antenna operating at 2.4 GHz (Figure 16a). The sensing mechanism relies on detecting shifts in the antenna’s resonance frequency, which are caused by changes in the dielectric properties of glucose/water solutions simulating different blood glucose concentrations.

The study tested the sensor with glucose solutions covering hypoglycemia (10–70 mg/dL), normoglycemia (80–110 mg/dL), and hyperglycemia (130–190 mg/dL). Figure 16b illustrates the linear regression analysis of resonant frequency shifts across three diabetic states. The results demonstrate a strong linear correlation between glucose concentration and the corresponding frequency shift, with a coefficient of determination (R^2^) of 0.96, indicating high predictive consistency.

**Figure 17:**
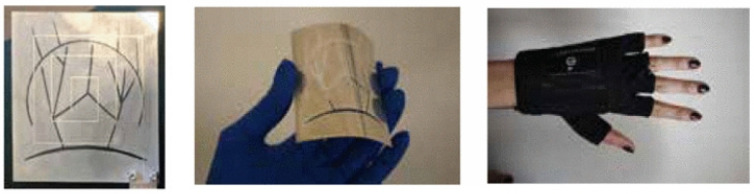
Slot antenna-sensor integrated inside a glove [111].

Ref. [111] developed an innovative antenna-sensor inspired by the structure of vascular anatomy, as illustrated in Figure 17. The device employs PET as its base material, with silver serving as the conductive component. A helical feed transmission line is integrated into the bottom layer, which significantly boosts the coupling efficiency between the feed line and the upper slot. This clever arrangement enables the activation of multiple slots, thereby enhancing the antenna’s ability to operate across multiple frequency bands.

The antenna-sensor demonstrated high accuracy within the diabetic glucose range (10–500 mg/dL). During in vitro testing at 1.1375 GHz, the correlation between the amplitude of S11 and the reference blood glucose level was 0.96. At 1.575 GHz, the correlation between the phase of S11 and the reference blood glucose level was 0.98. The clinical accuracy of the sensor was further substantiated by in vivo clinical trials, where 21 subjects conducted a total of 63 oral glucose tolerance tests, showing a 98 percent agreement between the reference glucose levels and sensor output.

In summary, flexible antenna-sensors have demonstrated clear applicability in non-invasive glucose monitoring, with recent studies validating their performance across both in vitro and in vivo settings. By leveraging wearable-compatible materials, textile-based fabrication, and anatomically inspired designs, these sensors have achieved high sensitivity within physiologically relevant glucose ranges. Their ability to integrate seamlessly into daily-use formats such as gloves or garments underscores their promise for real-world, continuous monitoring applications. Nevertheless, the current research remains limited in scope and scale, with most studies confined to preliminary prototypes. Further investigations are warranted to evaluate long-term performance, user variability, and integration with clinical standards.

## 4. Summary, Challenges, and Conclusions

### 4.1. Summary

Diabetes has become a significant global public health concern, with increasing prevalence and a rising burden on healthcare systems. The effective management of blood glucose levels is critical for preventing complications and reducing mortality. As a result, accurate, convenient, and continuous sensor technologies for blood glucose monitoring have become a cornerstone of diabetes care. This review explores a variety of sensor technologies, including invasive and non-invasive methods, to evaluate their potential in providing reliable and continuous glucose measurements. Among these, antenna-sensors have emerged as a promising alternative due to their low cost, compact design, and suitability for integration into wearable devices.

This review particularly focuses on the research comparing rigid and flexible antenna-sensors. Rigid antenna-sensors, typically made from metals, are known for their high accuracy and stability in detecting glucose levels. However, their inflexibility and bulkiness limit their practicality for wearable applications, where comfort and mobility are crucial. On the other hand, flexible antenna-sensors, constructed from materials such as textiles, polymers, and conductive fabrics, offer significant advantages in terms of comfort, wearability, and seamless integration with the human body. These sensors are ideal for non-invasive continuous, real-time blood glucose monitoring, as they allow for prolonged use without discomfort and can be easily incorporated into everyday clothing, making them well-suited for managing diabetes on a daily basis.

The development of flexible antenna-sensors for glucose monitoring represents a significant breakthrough in wearable medical devices, with profound implications for diverse populations. For diabetic patients, non-invasive and continuous glucose monitoring can significantly enhance the quality of life, optimize disease management, and reduce long-term healthcare costs. For at-risk individuals, real-time monitoring enables early detection and personalized health management, potentially delaying or preventing disease progression. For healthcare systems, real-time data transmission improves the diagnostic efficiency, advances precision medicine, and alleviates the societal burden associated with diabetes-related complications. At a broader societal level, this technology promotes healthy aging and reduces healthcare and economic pressures.

### 4.2. Challenges

Critical areas and obstacles are anticipated in future studies on adaptable antenna-sensors for non-invasive glucose tracking, including the following:

1. Mitigating the influence of environmental factors (e.g., humidity, temperature fluctuations) and human factors (e.g., mechanical stress from washing, sweat-induced corrosion) on antenna-sensor performance.
2. Enhancing the precision and efficiency of the current fabrication techniques.
3. Incorporating flexible antenna-sensors into everyday clothing while ensuring biocompatibility and long-term durability.
4. Investigating new yarns and conductive textiles with lower resistivity or higher conductivity to improve antenna-sensor performance.

### 4.3. Conclusions

Flexible antenna-sensors represent the convergence of electromagnetics, materials innovation, and personalized healthcare, offering a promising alternative to traditional glucose monitoring technologies. This review has demonstrated that although rigid antenna-sensors provide reliable electromagnetic performance, their lack of structural flexibility restricts their suitability for continuous, user-friendly applications. In contrast, flexible antenna-sensors have the structural adaptability and integration potential required to support non-invasive, real-time glucose tracking in everyday life.

However, their transformative potential extends beyond mere mechanical flexibility, facilitating a fundamental shift from episodic, reactive measurement to continuous, proactive metabolic monitoring. Realizing this vision requires overcoming closely related technical and physiological challenges, such as maintaining the signal fidelity during movement, ensuring long-term material stability, addressing inter-user variability, and embedding robust algorithmic interpretation. These challenges are not merely incremental but are system-level constraints that define the frontier of wearable biomedical sensing.

As antenna-sensor research evolves from simulation to real-world deployment, flexible architectures could catalyze a new paradigm in diabetes care—one that is not only non-invasive and clinically relevant but also seamless, invisible, and responsive to the demands of daily life.

## Figures and Tables

**Table 1:** Estimated number of people with diabetes (aged 20–79), in thousands [14].

Region	2000	2021	2030	2045
Africa	2532.9	23,633.9	33,446.0	55,254.4
Europe	22,373.1	61,425.1	67,000.0	69,000.0
Middle East and North Africa	17,007.6	72,671.9	95,000.0	135,700.0
North America and Caribbean	21,375.1	50,547.0	57,000.0	63,000.0
South and Central America	8553.3	32,497.1	40,000.0	49,000.0
South-East Asia	34,882.2	90,204.5	113,300.0	151,500.0
Western Pacific	44,097.9	205,640.2	238,300.0	260,200.0

**Table 2:** Commonly used rigid substrates in antenna-sensor design.

Material	Relative Permittivity	Advantages	Limitations
FR-4 (Flame Retardant 4)	4.4	Cost-effective and widely available; mechanically robust; compatible with standard PCB fabrication processes.	High dielectric loss; limited suitability for microwave and millimeter-wave applications.
Rogers Laminates (e.g., RO4003C, RO4350B)	2.2–10	Low dielectric loss; stable dielectric constant; superior thermal and mechanical stability.	Higher cost; requires specialized manufacturing processes; slightly lower mechanical strength.
Ceramic (e.g., Alumina, LTCC)	>10	Extremely low dielectric loss; stable high dielectric constant; high thermal stability; ideal for compact, high-frequency applications.	High fabrication costs; mechanically fragile; limited availability for rapid prototyping.
PTFE (Polytetrafluoroethylene)	2.1	Low dielectric loss; stable dielectric constant; excellent chemical and thermal resistance; preferred for precision RF applications.	High manufacturing cost; complex processing requirements; poor mechanical rigidity; susceptible to dimensional instability.

Note: The materials listed in Table 2 represent the most commonly used rigid substrates found in previously published studies on non-invasive glucose monitoring using rigid antennas. While other materials, such as glass and silicon, are also potential candidates, they are not included here for the sake of brevity.

**Table 3:** Commonly used rigid conductive materials in antenna-sensor design.

Material	Conductivity (S/m)	Advantages	Limitations
Copper	5.96 × 10^7^	High electrical conductivity; cost-effective; good mechanical strength and ductility.	Susceptible to oxidation; requires protective coatings or encapsulation in harsh environments.
Silver	6.3 × 10^7^	Highest electrical conductivity; excellent reflectivity and thermal conductivity; low contact resistance.	High cost; susceptible to tarnishing; softer and less mechanically robust.
Aluminum	3.5 × 10^7^	Lightweight; cost-effective; natural corrosion resistance.	Lower electrical conductivity; oxide affects electrical connections; brittle.
Gold	4.1 × 10^7^	Resistant to oxidation and corrosion; excellent biocompatibility.	Expensive; soft and prone to mechanical wear; higher density.

Note: The materials listed in Table 3 represent the most commonly used rigid conductive materials found in previously published studies on non-invasive glucose monitoring using rigid antennas. While other materials, such as nickel and indium tin oxide, are also potential candidates, they are not included here for the sake of brevity.

**Table 5:** Commonly used flexible conductive material in antenna-sensor design.

Material	Conductivity (S/m)	Advantages	Limitations
Adhesive copper, copper tape, and copper cladding	5.96 × 10^7^	Excellent electrical conductivity; high mechanical strength; stable under environmental conditions.	High density; prone to oxidation; limited stretchability; additional processes required.
Silver nanoparticle inks	10^5^ − 10^6^	Superior conductivity; low-temperature sintering; oxidation-resistant.	High cost; complex sintering process; limited stretchability.
Copper nanoparticle inks	10^4^ − 10^5^	Lower cost compared to silver; suitable for large-area printed electronics.	Prone to oxidation, requires protective coatings or reducing agents.
PANI and PPy	10^0^ − 10^2^	Lightweight, flexible; chemically tunable, environmentally stable.	lower conductivity than metals; mechanical fragility; conductivity varies with the doping level.

Note: The materials listed in this table represent commonly used examples in the literature and do not constitute an exhaustive list of all available material options.

**Table 6:** Dielectric properties of common commercial textiles [82].

Nonconductive Fabric	Dielectric Constant	Dielectric Loss
Cotton	1.6	0.04
Felt	1.215	0.016
Silk	1.75	0.012
Jeans	1.7	0.025
Fleece	1.17	0.0035
Denim	1.6	0.05

Note: The materials listed in this table represent commonly used examples in the literature and do not constitute an exhaustive list of all available material options.

**Table 7:** Commonly used e-textile materials in flexible antenna-sensor design.

	Zelt	Pure Copper Taffeta Fabric	Shieldit Super
Conductivity (S/m)	1.749 × 10^5^	2.5 × 10^5^	6.67 × 10^5^
Surface Resistance (Ω)	0.05	0.05	1

Note: The materials listed in this table represent commonly used examples in the literature and do not constitute an exhaustive list of all available material options.

## Data Availability

No new data were created.
